# Functional assessment of time course microarray data

**DOI:** 10.1186/1471-2105-10-S6-S9

**Published:** 2009-06-16

**Authors:** María José Nueda, Patricia Sebastián, Sonia Tarazona, Francisco García-García, Joaquín Dopazo, Alberto Ferrer, Ana Conesa

**Affiliations:** 1 Department of Statistics and Operation Research, University of Alicante, Ctra. San Vicente del Raspeig, S/N 03690 Alicante, Spain; 2 Bioinformatics and Genomics Department, Centro de Investigaciones Príncipe Felipe, Avda. Autopista Saler 16, 46012 Valencia, Spain; 3 Department of Applied Statistics and Operations Research, Universidad Politécnica of Valencia, Cno. vera s/n, Edifico 7A, 46022 Valencia, Spain; 4 Functional Genomics Node (INB), Centro de Investigación Príncipe Felipe (CIPF), Valencia, Spain; 5 CIBER de Enfermedades Raras (CIBERER), ISCIII, Spain

## Abstract

**Motivation:**

Time-course microarray experiments study the progress of gene expression along time across one or several experimental conditions. Most developed analysis methods focus on the clustering or the differential expression analysis of genes and do not integrate functional information. The assessment of the functional aspects of time-course transcriptomics data requires the use of approaches that exploit the activation dynamics of the functional categories to where genes are annotated.

**Methods:**

We present three novel methodologies for the functional assessment of time-course microarray data. i) maSigFun derives from the maSigPro method, a regression-based strategy to model time-dependent expression patterns and identify genes with differences across series. maSigFun fits a regression model for groups of genes labeled by a functional class and selects those categories which have a significant model. ii) PCA-maSigFun fits a PCA model of each functional class-defined expression matrix to extract orthogonal patterns of expression change, which are then assessed for their fit to a time-dependent regression model. iii) ASCA-functional uses the ASCA model to rank genes according to their correlation to principal time expression patterns and assess functional enrichment on a GSA fashion. We used simulated and experimental datasets to study these novel approaches. Results were compared to alternative methodologies.

**Results:**

Synthetic and experimental data showed that the different methods are able to capture different aspects of the relationship between genes, functions and co-expression that are biologically meaningful. The methods should not be considered as competitive but they provide different insights into the molecular and functional dynamic events taking place within the biological system under study.

## Background

Microarray time-course experiments have gained popularity in recent years to address the study of biological phenomena where the dynamics of gene expression is of relevance. In contrast to classical control-case studies, where basically two conditions are compared, time series experiments encompass investigations of diverse nature and complexity. Studies may relate to developmental processes with a large number of sampling points (e.g. [1]), or to stimuli-response experiments where transcriptome changes are assessed in a short time span and may include multiple treatments (e.g. [2]), or may try to capture cyclic variations of gene expression (e.g. [3]). Moreover, samples might be destroyed by the sampling process or be taken from the same individuals along the time component. This results in microarray time-course data being classified as either *short *(up to 5–6 time points) or *long *(from 6–7 time points) series, *single *(one treatment or tissue) or *multiple *(more than one treatment or tissue) series, and *longitudinal *vs. *independent *depending if samples are blocked by an individual effect or are not related. A significant number of statistical methods have been published as microarray time-course experiments that have been expanded to address the analysis of this type of data. Many of the developed algorithms consider the clustering of serial data. Proposed strategies include the use of Gaussian mixed models [4], Bayesian models [5], Hidden Markov Models [6], B-splines [7,8], and Fourier Series [9] to model and cluster long series data, while more *ad-hoc *algorithms have been developed for short series [10,11]. Another important block of methodologies are those that pursue the identification of time-associated differentially expressed genes (d.e.g.'s). In this category we find back the B-spline approach [7,12] a multivariate adaptation of the empirical Bayes test [13] to specifically treat longitudinal data [14] and some ANOVA and regression-based models for short series [15-18]. Finally, Conesa and co-workers presented two methods well suited to independent, multiple series data based either on step-wise regression or singular component analysis [19,20].

In all of these approaches statistical analysis focused on modeling gene expression and identifying those genes with a relevant variation pattern. This orientation, though valid and useful, solves only one (frequently the first) requirement to understand transcriptomics changes from any kind of microarray experiment. In most cases, the analysis proceeds through the identification of cellular processes and functions which are represented by the gene selection, i.e. genes are identified by their functional role and the question is then which functional modifications can be derived from the observed gene regulation. The incorporation of functional information into data analysis is normally obtained by the use of functional annotation databases that define and assign function labels to known genes. The most widely used functional annotation scheme is the Gene Ontology (GO) [21], which characterizes genes for their molecular functions (MF), cellular locations (CC) and involved biological processes (BP), but others such as the KEGG metabolic pathways [22], transcription factor targets [23] or Interpro functional motifs [24] can also be employed for specific biological questions. This functional assessment aspect is traditionally handled in microarray data analysis via the so-called enrichment analysis: the list of significant genes is interrogated for over (and/or under) abundance, as compared to the entire genome represented in the array of the considered functional categories. In time-course microarray data, this strategy could be similarly followed for the set of time-dependent differentially expressed genes (for example, as provided in the time course module of the GEPAS suite, [25]), or for the distinct clusters into which this gene selection can be divided (available in STEM package, [26]). As a matter of fact, gene enrichment analysis is very often used to validate the results of a gene selection or a clustering strategy [27,28].

This strategy for the functional evaluation of differential gene expression has a number of limitations [29]. Firstly, the functional enrichment analysis is greatly dependent on the definition of an arbitrary threshold for significance and gene selection, and eventually on the clustering strategy of choice. The threshold aspect was overcome in two class experiments through the Gene Set Analysis approaches (GSA), which evaluate functional enrichment over a rank rather than a selection of genes [30-33]. To our knowledge, no equivalent approach is yet available for time series data. Secondly, functional assessment is done after gene selection and therefore does not allow for a direct evaluation of expression changes as gene functions, which might obscure relationships between functional categories and ignore significant sub-patterns of variations within the functional class.

In this paper we have set out to address the problem of the functional assessment of gene expression in time series data in an alternative manner. We have developed and tested three distinct strategies which respond differently to the various concerns mentioned above. The proposed methods derive from previous methodologies developed in our group for the analysis of short, multiple series data which follow a gene-centric orientation: the maSigPro [19], a two-step regression approach, and the ASCA-genes [20], a multivariate method that combines ANOVA decomposition with Singular Component Analysis. In this study, we first assess the fully correlated nature of the functional category in a modification of the maSigPro methodology to directly model the combined expression of genes belonging to the same functional class (maSigFun). In a second approach, we consider the possibility of different patterns of coordinative time-dependent gene expression variation within the functional class and the selection of those with a significant change (PCA-maSigFun). Finally, we develop an adaptation of the GSA strategy to time series by identifying the main variation patterns in the dataset and rank genes according to their correlation to such patterns (ASCA-functional).

We have used both synthetic and experimental datasets to assess the different methods. Simulated data provides a means of understanding the working of the methodologies while experimental data offers insights into the biological relevance of the strategies. Furthermore, we provide a comparison with other available methods. Algorithms were implemented in the R language and are available at .

## Materials and methods

### The proposed methods

#### maSigFun

This methodology derives from maSigPro, a regression-based approach for the analysis of multiple series time-course microarray data [19]. The maSigPro method follows a two-stage regression strategy to model gene expression and select differential expressed genes: the first step uses a generic polynomial model to spot responsive genes, while the second applies step-wise regression to reveal the patterns of significant differential time profiles.

**Figure 1:**
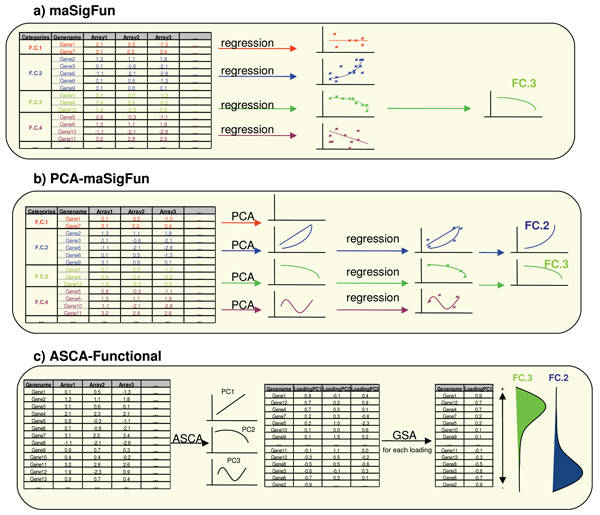
**Schematic representation of the proposed methods**. a) maSigFun fits a regression model for each gene expression submatrix defined by the genes annotated to a given functional class (FC.1 to 4 in scheme). Significant classes are obtained by the maSigPro method (FC.3). b) PCA-maSigFun obtains a PCA model for the gene expression submatrix defined as in maSigFun and extracts a number of components that collect non-random variation. Generally 0 (FC.1) to 2 (FC.2) components are extracted for each functional class. A regression model is then fitted to the scores vector of extracted components to select function-defined patterns with a significant association to time (FC.2 and FC.3). c) ASCA-functional applies ASCA-genes to identify principal patterns of variation associated with time and time × treatment experimental factors (PC1 to 3 in scheme). Genes are ranked by loading value in each PC, and GSA analysis is applied to each loading value-ordered gene list to identify a functionally related block of genes associated with the principal patterns of variation (FC.2 and FC.3).

The adaptation of maSigPro to consider functional information -maSigFun- is quite straightforward: the regression model is not fitted gene-wise as in maSigPro, but to the data matrix composed the expression values of all genes belonging to the functional class, thus one regression model is fitted to each functional category. In this approach individual genes are considered as different observations of the expression profile of the class. As genes belonging to the same class may show different basal expression levels and this may negatively influence the estimation of model parameters, expression data is standardized gene-wise to better capture the correlation structure within the functional group. After this transformation, statistical analysis proceeds as in regular maSigPro (Figure 1a). The expected result is that significant functional classes are those whose genes change their expression along time in the same manner, i.e. a high level of co-expression is present within the functional class.

#### PCA-maSigFun

In this strategy we consider that a functional block might display not only one but several patterns of coordinative gene expression. These distinct patterns are extracted by following a strategy similar to that proposed by [34] to directly link gene function to the phenotype. Basically, the strategy applies Principal Component Analysis (PCA) to the gene expression matrices composed of all genes belonging to the same functional class. PCA modeling will dissect orthogonal, time-dependent, transcriptional patterns contained in the class and a number of those will be selected. The selection criterion implemented in the PCA-maSigFun method follows the rationale of retaining patterns that represent non-random variation. Considering the general assumption held in transcriptomics analysis, that of global invariability in gene expression, a good estimate of noise-level variation would be the mean gene variance across the complete dataset. Therefore, for each functional-class associated PCA, selected components are those having a normalized explained variance above this mean gene variance value. The scores vector of each component depicts an expression pattern that corresponds to a correlated gene subset of the functional class and can be taken as transformed expression values for that subset. All thus-obtained scores vectors are collected into a matrix of function-labeled "synthetic genes" which is then subjected to regular maSigPro for regression-based statistical analysis. Selected features will therefore correspond to defined function patterns that show a significant association with time (Figure 1b). Once significant functional features are obtained, the question is how individual genes relate to these significant patterns. This information can be obtained by analysing the gene loadings in each PCA model. Genes with a high absolute loading value in a given selected component will have an important contribution to the associated profile and therefore can be considered as members of the gene subset that defined that correlated pattern of the class. Genes with a low absolute loading value will not correspond to this subset. In the current PCA-maSigFun implementation, a value for loading cutoff is derived by bootstrapping over the whole dataset to create a null loadings distribution across all functional classes and defining an arbitrary threshold (typically the 95% percentile) to declare a gene as significantly contributing.

#### ASCA-functional

The ASCA approach was developed by [35] to analyze high dimensional data obtained from replicated, multi-factorial designed experiments. [20] adapted this methodology to transcriptomics analysis by incorporating a strategy for gene selection (ASCA-genes). Basically, ASCA couples ANalysis Of VAriance (ANOVA) decomposition to Singular Component Analysis (SCA) to study correlation patterns associated with the experimental factors of interest. In the case of transcriptomics time series data, ASCA extracts gene expression submatrices where only signals associated with time and/or treatment are retained. SCA applied to these submatrices identifies patterns of co-expression across genes where noise and possible co-variate effects have been removed. ASCA analysis therefore provides a PCA submodel for each experimental factor-time, treatment and the interaction – that encompasses all genes in the dataset and collects most of the variability associated with each experimental factor. In ASCA-functional these models are used to create ranks of genes that can be subjected to GSA analysis. In this sense, the third proposed approach can be considered as an adaptation of GSA methods to situations where not only two, but more experimental conditions are involved, as is the case of (multiple series) time course data. In two-class data, genes are ranked according to a measure of differential expression such as fold change, a *t*-statistic or a similar statistic. Enrichment analysis is performed along this rank by assessing the differential distribution of each functional block along the ranked gene list. In the case of ASCA-functional, ASCA-genes is first applied to create PCA submodels associated with each experimental factor. Similarly to the previous method, the genes loadings at each component of each submodel are a measure of the similarity of each particular gene expression profile to the pattern depicted by the component of the submodel. Genes with high positive loadings will mostly follow the pattern indicated by the component; genes with high negative loadings follow an opposite pattern, while genes with loadings close to zero do not resemble the behavior represented by the principal component. Those badly modeled genes are identified in ASCA-genes by their high SPE [20] and are assigned a loading value of 0. The gene loadings therefore offer a way to rank genes according to specific patterns of variation which correspond to biological phenomena. This ranking can then be subjected to GSA analysis. In our particular implementation, ranked lists are analyzed by the partitioning method FatiScan [30,33] to identify functional categories associated with specific time patterns (Figure 1c).

### Datasets

Synthetic and experimental datasets were used to assess the proposed methods. Synthetic data was designed to depict different scenarios of co-expression while the experimental sets reflect two microarray studies involving different probe sizes and biological systems.

#### Synthetic datasets

Two simulation studies were designed to evaluate the effect of class size and the percentage of co-expressed genes in the identification of time-course by changing functional categories. Both studies use the same primary data structure. The hypothetical experiment contained two series (Control and Treatment) and three time-points (0, 1 and 2). Synthetic datasets consisted of a total of 10,000 genes in study A and several sizes in study B, distributed in 250 classes from which 225 classes contain only flat genes and 25 classes include at least one differentially expressed gene. Modeled responsive genes follow one of four possible patterns of expression: 1) Flat profile for control and continuous induction for treatment, 2) Flat profile for control and continuous repression for treatment, 3) Flat profile for control and transitory induction for treatment and 4) Flat profile for control and transitory repression for treatment. In all of the 25 classes with some non-flat genes only one of the four patterns is present, meaning that all changing genes in the class follow the same profile, have a positive correlation and could be regarded as "co-expressed". In each individual simulation, noise was introduced into the datasets by adding to the defined profiles random values taken from a normal distribution N(0, 0.01).

The first simulated study (A) analyzes how the percentage of co-expressed genes within the functional class affects the identification of the category. In this study, functional classes varied in size (number of genes), taking values from 5, 10, 30, 55 and 100. Seven different datasets were created in this study, each of them with a different percentage of co-expressed genes (20, 30, 40, 50, 60, 70 and 80%) for all of the 25 non-all-flat classes present in the dataset (Table 1). For example, dataset A-40 has 10,000 genes distributed in 250 classes of different size from which 25 classes all have 40% of genes which follow the same changing profile and 60% of the genes that are flat. In the remaining 225 classes of dataset A-40, 100% of the genes are invariant. Fifty simulations were run for each of the seven proportion levels.

**Table 1:** Summary parameters used in simulation studies.

	**Simulated Study A**	**Simulated Study B**
**Number of categories**	250	250
**no. categories with d.e.g**.	25	25
**Scenarios analised**	7 (% d.e.g.'s)	12 (% d.e.g.'s (3) × no.genes categories (4))
**% d.e.g.'s within category**	20,30,40,50,60,70,80	30,50,70
**no. genes in each category**	In each scenario, categories have different number of genes, systematicaly taken from 5, 10, 30, 55, 100	In each scenario, all categories have the same number of genes. Different cases: 5,10,50,100
**Total number of genes**	1000	1250, 2500, 12500, 25000 (250 × no. genes in each category)

d.e.g.'s: differentially expressed genes

In the second simulated study (B) we evaluated the effect of the class size. Here, 4 × 3 datasets were created, each of them having a fixed value for the class size (5, 10, 50 and 100) and a fixed value for the percentage of genes with change (30, 50, and 70%) (Table 1). For example, dataset B-50-70 contains 250 classes of size 50 (12500 genes), from which 25 classes have 35 genes with a defined changing profile and 15 flat genes, while the remaining 225 classes of dataset B-50-70 all have 50 genes flat. Again, 50 simulations are run for each size and proportion levels.

#### Experimental datasets

Three experimental datasets, representing different technological platforms and array sizes, were selected for the evaluation of the methodologies on real data. The first dataset corresponds to the toxicogenomics study by [36] where the transcriptome response in rat liver to increasing doses of the drug bromobenzene (BB) is studied. In this study 2–6 rats were sacrificed after 6, 24 or 48 hours of drug exposure to extract liver mRNA which was then labeled and hybridized to a custom cDNA using a dye-swap design with a common reference. The dataset consists of 3 time points, 5 series (HIgh, LOw and MEdium BB levels, UnTreated and Corn Oil vehicle controls) and 2,665 genes. The second dataset collects the transcriptional response to three different abiotic stressors (Salt, Cold and Heat) in potato measured on the NSF 10 k potato array [37]. Also a common reference design is used in this case. The dataset has 4 series (3 treatments plus one Control), 3 time points and three replicates per experimental condition. The third experimental dataset is taken from a microarray platform evaluation study for which the indole-acetic acid (IAA) hormone treatment in *Arabidopsis thaliana *is used to assess the performance of the 23 K Affymetrix ATH1 GeneChip [38]. This dataset counts with three time points (0 h, 1 h and 3 h after treatment) and two levels of hormone administration, 0.1 uM IAA and 1.0 uM IAA. There are between 6 and 4 replicates per experimental condition.

### Comparison to other methods

We compared our results on experimental data with two different methodologies available at the time of research. The methods were chosen because they have ready-to-use implementations or were specifically described for the functional analysis of time series data.

We used the STEM software available at . STEM implements the algorithm described in [39] for clustering short time-series gene expression data. The method works by assigning genes to a predefined set of model profiles that capture the potential distinct patterns that can be expected from the experiment and assessing the significance of these patterns. STEM is fully integrated with the Gene Ontology database supporting GO category gene enrichment analyses for sets of genes belonging to the same cluster. Secondly, we compared our results with the strategy described in [40] for finding blocks of functionally related genes in experiments which display an autocorrelation between successive points. Basically the strategy computes the difference between each time point and the zero time for all genes in the experiment to create a matrix of pair-wise differences. Each column in the matrix is then ordered by the magnitude of the difference to generate as many gene ranks as time points. Each ranked list is then individually subjected to GSA, as implemented in the FatiScan program [33] and functional results are jointly evaluated.

## Results

### Simulation studies

For either simulation study, fifty datasets of each type were generated and analyzed with the three proposed methods. For each observation, the identified categories were recorded and values of true positives (considering the 25 non-all-flat classes as "true positives"), values of false positives (FP), false negatives (FN), sensitivity (proportion of actual positives which were correctly detected) and specificity (proportion of negatives which were correctly identified) were computed. In all methods the significance threshold was set at 0.05 false discovery rate (FDR).

#### maSigFun

In the case of maSigFun, analysis recall statistics were calculated at different values of the R2 parameter since this was expected to have a great influence on the results. The R2 or goodness of fit indicates how well the model fits the data and therefore reflects the coherence within the observations. Previous studies with maSigPro indicated that a cut-off value of 0.6 would be appropriate for the selection of d.e.g.'s in time-course microarray data [19]. In this study, four levels of R2 , 0, 0.4, 0.6 and 0.8 were evaluated. Results are presented in Additional file 1.

**Figure 2:**
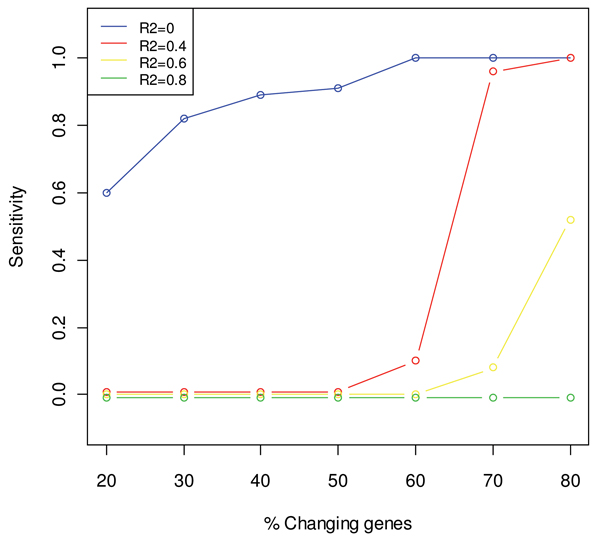
**Results of simulation study A with the maSigFun method**. Changes in sensitivity with the percentage of co-expressed genes in the class at four values of the goodness of fit R2 of the regression models. Data points correspond to the mean value of 50 simulations. Confidence intervals were omitted due to their negligible size.

The *in silico *analysis revealed that the maSigFun methodology is sensitive at identifying functional classes with a high proportion of changing genes (70%) when a moderate R^2 ^cut-off (0.4) is imposed. At higher R2 sensitivity drops, while the consequence of releasing the R2 filter (R2 = 0) was that functional classes with a low proportion of regulated genes (20%–30%) could also be selected (Figure 2). In all cases, the rate of false positives is under control and specificity remains high (see Additional file 1).

**Figure 3:**
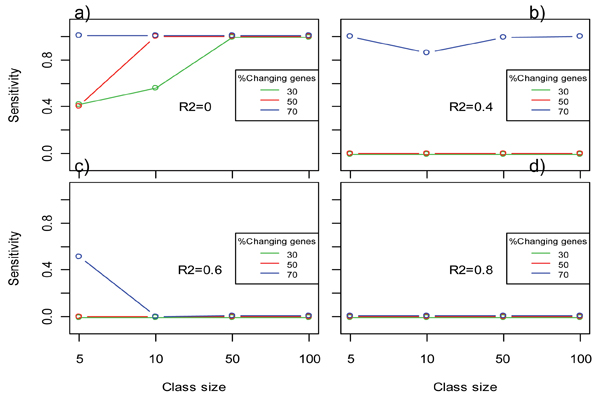
**Results of simulation study B with the maSigFun method**. Changes in sensitivity with the size of the class at three levels of percentage of changing genes (co-expression) in the class. One plot is provided for each level of the goodness of fit R2 of the regression models. Data points correspond to the mean value of 50 simulations. Confidence intervals were omitted due to their negligible size.

Regarding class size, simulation study B showed that this factor is of little relevance when a sufficient level of co-expression and R2 cut-off value are used, as the sensitivity of the method is more dependent on the amount of regulated genes in the class (Figure 3, panels b), c) and d)). However, when functional classes have a lower level of co-expression and a permissive R2 is used, maSigFun revealed a dependency on the class size, because the method is more sensitive for classes with a large number of members (Figure 3, panel a)). Again, specificity was high in all cases (see Additional file 1).

Taken together, the simulation analysis showed that maSigFun is effective at identifying those functional classes for which a relative high level of gene expression coherence is present regardless of the number of genes annotated to the class.

#### PCA-maSigFun

The simulation analysis for the PCA-maSigFun resulted in sensitivity and specificity values close to one in all scenarios and dataset types (see Additional file 1), indicating that the method basically identifies any functional class with at least 20% of changing genes, regardless of it size, and also that the methodology is robust for the occurrence of false discoveries. This result is not surprising, since the specific property of the method is the ability of extracting gene expression sub-patterns within each class and the positive selection of the functional class occurs by identifying the correlated profile.

#### ASCA-functional

**Figure 4:**
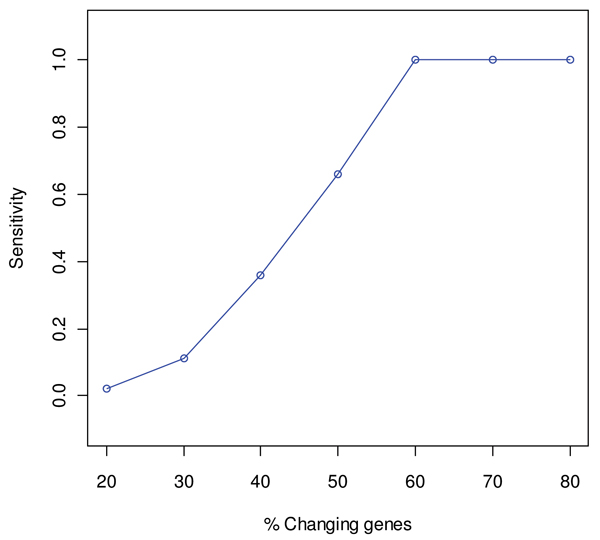
**Results of simulation study A with the ASCA-functional method**. Changes in sensitivity with the percentage of changing genes (co-expression) in the functional class. Data points correspond to the mean value of 50 simulations. Confidence intervals were omitted due to their negligible size.

As only one pattern of variation was modeled in each synthetic functional class, ASCA was applied with only one component in the submodel, capturing time and treatment effects, denoted as "submodel b+ab" in the ASCA-genes paper [20]. Genes were ranked according to the loading value of this single component, leading to one FatiScan analysis per synthetic dataset. The *in silico *study for ASCA-functional also showed interesting results. Simulation study A revealed a turning point for sensitive detection at a percentage of changing genes of 60% (Figure 4). This result is in agreement with the nature of the GSA strategy since the asymmetric distribution along the ordered gene list of the genes annotated to a given class is expected to occur when the percentage of genes associated with the biological phenomenon captured by the ASCA component is above half of the class size. On the other hand, simulation study B indicated that the size of the class does not affect sensitivity of detection which is merely dependent on the inner co-expression level of the class. Full specificity was obtained for all dataset types in both studies (Additional file 1).

### Experimental datasets

The different functional assessment methods were applied to the analysis of two different experimental datasets. Since in real datasets the true differentially expressed genes are not known, recall statistics cannot be calculated. Therefore results were evaluated in terms of number of functional classes detected and biological coherence of the selection. The Gene Ontology was used a functional classification scheme. The set of GO terms characterizing each dataset was obtained by fetching GO information from public databases, completing annotation with the Blast2GO software [41], constructing the Direct Acyclic Graphs (DAGs) of each GO branch -BP, MF and CC- and obtaining all nodes in the graph. This set of terms was then refined by removing annotation redundant terms. A GO term was considered annotation redundant if it has the same set of annotated genes as any of its child terms.

#### Toxicogenomics dataset

In this study, three increasing doses of the drug bromobenzene were tested for their toxic effects on rat liver. The original analysis of the data showed that most marked effects on the transcriptome were provoked at high BB doses and 24 hours post-administration. Also important but more moderate were the effects of medium dose and later time points. The 2,665 probes contained in the rat chip were annotated to a total of 967 BP, 534 MF, and 243 CC non redundant GO terms (Table 2a).

**Table 2:** Functional analysis results for experimental dataset. Number of functional terms in each of the three GO branches present in the original dataset, after removal of redundant annotations and selected after analysis with each of the proposed methods. A) Toxicogenomics study, B) Potato Stress study, C) Arabidopsis IAA treatment study.

**A) Toxicogenomics study**							
					
			**SELECTIONS**				
**GO category**	**Original Annotation**	**Non redundant Annotations**	**maSigFun**	**PCA-maSigFun**	**ASCA-Functional**	**STEM**	**Pair-wise**
		
**BP**	1828	967	7	33	15		
**MF**	992	534	8	15	20		
**CC**	398	243	0	10	8		

		**total**	**15**	**58**	**43**	**0**	**49**

**B) Potato Stress study**							
					
			**SELECTIONS**				

**GO category**	**Original Annotation**	**Non redundant Annotations**	**maSigFun**	**PCA-maSigFun**	**ASCA-Functional**	**STEM**	**Pair-wise**

**BP**	2444	780	23	258	116		
**MF**	943	431	21	141	29		
**CC**	369	203	14	48	46		

		**total**	**58**	**447**	**191**	**0**	**46**

**C) Arabidopsis IAA study**							
					
			**SELECTIONS**				

**GO category**	**Original Annotation**	**Non redundant Annotations**	**maSigFun**	**PCA-maSigFun**	**ASCA-Functional**	**STEM**	**Pair-wise**

**BP**	2640	1120	3	60	201		
**MF**	1769	694	8	24	45		
**CC**	499	260	0	8	61		

		**total**	**11**	**92**	**307**	**46**	**284**

The three analysis approaches provided semantically related results but with very different levels of specification (Additional file 2).

**Figure 5:**
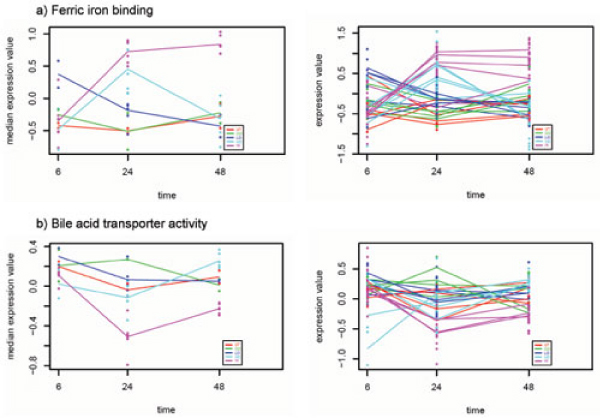
**Gene expression profiles for two significant representative GO categories obtained by maSigFun analysis of the Toxicogenomics dataset**. a) *Ferric iron binding *category induced by high Bromobenzene and b) *Bile acid transporter activity *category repressed by high Bromobenzene. On the left panel, the median value of the functional class is plotted while the right panel shows the expression profiles of all genes annotated in the class. Treatments are labeled by color: pink HI, light blue ME, dark blue LO, green CO and red UT.

maSigFun analysis identified 7 BP, 8 MF and 0 CC categories (Table 2a and Additional file 2) as significant at a FDR level of 0.05 and R2 of 0.3. More restrictive values for the R2 parameter failed to give any significant result. Functional categories included *heme oxidation*, *cell-aging*, *caspase activation via cytochrome c oxygenase*, *ferric ion binding*, *rRNA binding *and *plasminogen activator activity*, induced by BB administration, and *bile acid transporter activity*, *oxidoreductase activity*, *retinol binding *and *long-chain-fatty acid-CoA ligase activity*, repressed by high BB (Figure 5). Interestingly, selected categories had between 4 and 6 annotated genes and a mean inner correlation value (computed as the mean value of all pair-wise Pearson correlations of the expression profiles of the genes annotated to the class) of 0.6 ± 0.1. This measure of class coherence is close to the critic value of 70% percentage of co-regulated genes obtained in the simulation studies for efficient selection by maSigFun.

**Figure 6:**
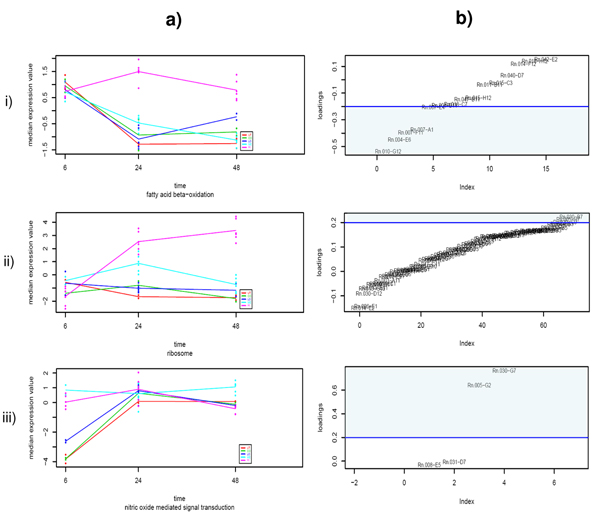
**Score vs Loading analysis of PCA-maSigFun results on the Toxicogenomics dataset**. a) Score profiles for three representative GO-components. b) Loading plot (gene contributions) for the same GO-components, genes labeled by their array ID. Blue lines indicate the threshold for significant contribution obtained by re-sampling (see methods).

**Figure 7:**
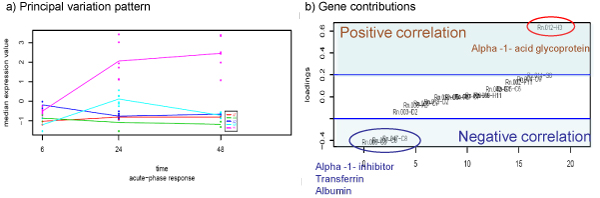
**Principal variation pattern of *acute-phase response *GO category in Toxicogenomics dataset analyzed by PCA-maSigFun**. a) Scores plot reveals the profile of the GO-component. b) Loadings plot show gene contributions. Threshold for significant contribution are indicated by blue line. Names of positively correlated and negatively correlated significant contributing genes are indicated.

Analysis by PCA-maSigFun provided a much richer repertoire of functional classes. GO-based PCA transformation of gene expression data compressed transcriptional information into function-associated transcriptome patterns ("synthetic genes", referred here as "GO-components"). In most cases one or two GO-components were obtained per GO term and only in very generic classes, such as *translation *or *ribosome*, up to 3 patterns of correlated behaviors were extracted. maSigPro analysis on the matrix of these new functional variables resulted in the identification of 33 BP 15 MF and 10 CC significant features (Table 2a and Additional file 2). Interpretation of these results is facilitated by plotting the PCA scores of each maSigPro significant GO-component along with the PCA loading of the annotated genes. In this way we can identify the gene expression patterns captured by the significant GO-component (Figure 6a) and locate the most contributing genes (Figure 6b), i.e. genes that most closely follow the pattern indicated by the GO-component either with a positive (+, gene loading greater than 0), or negative correlation (-, gene loading smaller than 0). Horizontal lines indicate the threshold for significant contribution of the gene to the GO-component pattern. The PCA-maSigFun approach identified 3 different patterns of expression: i) classes that show a peak of expression on high BB and 24 hours, ii) classes that also respond at 24 hours at medium BB and iii) classes that show a early (6 hrs) regulation for both high and medium BB (Figure 6). The first pattern was found for different GO terms pointing to processes as f*atty acid metabolism and oxidation *(-), *cell adhesion *(-), *amino acid metabolism *(-), *translation *(+,-) *microtubule organization *(+), *endopeptidase inhibitor activity *(-) and *vesicular fraction *(+). Functions associated with the second pattern include *translation *(+), n*egative regulation of cell proliferation *(+), *acute inflammatory response *(+,-), *xenobiotic metabolic process *(+,-), *signal transduction *(+,-), *biopolymer methylation *(-), *maintenance of localization *(+), *response to toxic compound *(+), *iron ion binding *(+,-), *exopeptidase activity *(+), *kinase activity *(+), *epoxide hydrolase activity *(+), *ribosome *(+,-). Finally, in the third pattern we found *cation homeostasis *(+), *nitric oxide mediated signal transduction *(+), *copper ion binding *(+) and *lysosome *(+). It is important to mention that, in most cases, only a subset of each GO term annotated genes showed significant contributions to the GO-component, indicating the predominant role of these genes in the determination of the pattern. In a few cases, corresponding to very general categories such as *translation *or *ribosome*, none of the annotated genes reach the threshold of significant contribution, but a continuum signal was observed, which would indicate a small but coordinated gene activity within the class. Finally, in some cases, such as *xenobiotic compound *and *acute-phase*, genes were observed that display either a positive or negative significant contribution to the component, which implies that coordination is present but with positively and negatively acting elements. For example, in the case of *acute-phase*, the *alpha-1-glycoprotein*, a positive acute phase protein, was found to have a significant contribution to the acute-phase GO-component pattern that represented gene expression activation with high BB at 24 h. Another three proteins, *alpha-1-inhibitor*, *albumin *and *tripsin*, known as negative acute-phase proteins [33], had significant but negative contributions to the GO pattern, which indicates an opposite pattern of expression (Figure 7). Therefore, this GO-component collects the induction of positive acute-phase proteins and the repression of negative acute-phase genes, suggesting a general activation of this cellular process.

**Figure 8:**
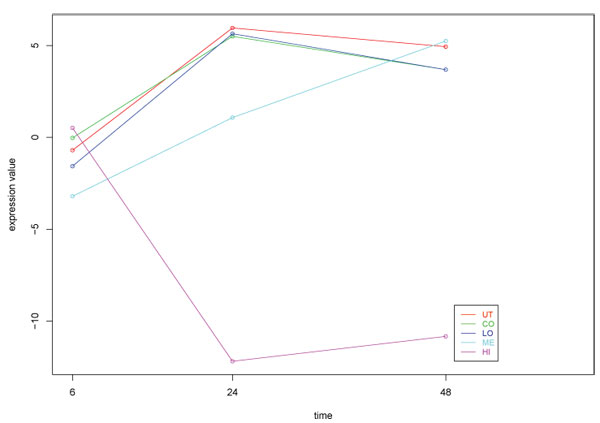
**Principal variation pattern in the Toxicogenomics dataset**. The pattern is captured by the first component of submodel b+ab (treatment + timextreatment) of ASCA-functional analysis. The plot shows the score values of this first component.

Finally the ASCA-functional method gave an intermediate result between the two previous approaches. Analysis by ASCA indicated three main independent patterns of variation within the transcriptomics signal. As in the other approaches, the first component, which collects 46% of the gene expression variability, represents the pattern of change (induction or repression) by high BB at 24 hours (Figure 8). The second component, with 10% associated explained variance, represents the change of medium BB at 24 hours. The third component (9% explained variance) captures the early responses at medium and high BB. As the first principal component represents mostly the toxicological response, this was the one subjected to FatiScan that resulted in the identification of 15 BP 20 MF and 8 CC significant features (Table 2a and Additional file 2). Significant processes included *ribosome*, *ferric ion binding*, *rRNA binding*, *energy *and *electron transport *at the upper end of the gene rank, indicating that these functions are positively correlated with the pattern provided by the first ASCA-genes component of submodel b+ab, i.e, induction by high BB at 24 h. GO terms such as *retinoic metabolic process*, *fatty acid beta oxidation*, *glutamine family amino-acid metabolism*, *oxidorreductase activity *were found significantly enriched at the bottom end of the gene rank, indicating their opposite correlated pattern of change.

#### NSF potato stress dataset

**Figure 9:**
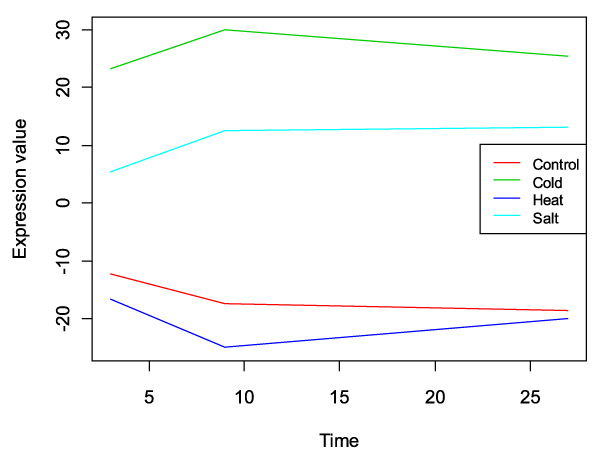
**Principal variation pattern in the Potato Stress dataset**. The pattern is captured by the first component of submodel b+ab (treatment + timextreatment) of ASCA-functional analysis. The plot shows the score values of this first component.

The Potato Stress dataset consists of three abiotic stress series (cold, heat and salt treatments) plus one control series measured along 3 time points on the NSF potato 10 k chip. In general, the three different approaches behaved in a similar fashion as in the toxicogenomics dataset although a much richer functional response was observed in this study. The major gene expression pattern within this dataset corresponds to the differential behavior of the cold and salt stresses with respect to the control and heat conditions. A differential regulation is observed between the two pairs of series already at 3 hours, peaking at 9 hours and maintained till the end of the experiment (Figure 9).

The number of functional classes obtained with each of the methods is shown in Table 2b and a complete list of all significant GO terms is provided in Additional file 2. maSigFun analysis gave the smallest amount of significant GO terms, which had on average 6.4 annotated terms and a mean inner correlation value of 0.63 ± 0.1. Significant functions corresponded to profiles of induction (+) or repression (-) of the class as a whole for the cold and salt stressors with respect to the control and heat conditions. Down-regulated processes included photosynthesis-related terms, *fructose metabolism*, *cell-wall modification*, *lateral root morphogenesis *and *reductive pentose-phosphate cycle*. Up-regulated processed referred to protein turnover, r*esponse to hypoxia *and *glucose stimulus*, *multi-drug transport*, *salicylic acid signaling pathway *and diverse enzymatic activities. PCA-maSigFun again gave a much richer view on cellular processed (447 selected GO terms) and highlighted additional functions such as *response to stress, chitinase activity, oxidoreductase activity*, *transmembrane transport*, *secretory pathway*, j*asmonic acid signaling *and *abscisic acid pathways*, among many others. Finally ASCA-functional analysis indicated the major pattern of variability as the difference between the cold and salt stresses on one hand and heat and control conditions on the other, this pattern 57% comprising of the variability contained in the dataset (Figure 9). FatiScan analysis on the gene loadings rank provided by this first component indicated as significant most of the processes revealed by the other methods, i.e., response to several stimuli, *protein synthesis and degradation*, diverse hormone signaling pathways, *lignin biosynthesis *associated with genes in upper rank positions; *photosynthesis*, *microtubule-based movement*, *RNA binding *and *lypoxigenase activity *as processed over-represented in bottom rank genes. Taken together, the results of the three different approaches reveal, at different levels of detail, the cellular response triggered by the treatments. While the heat stress does not seem to provoke, at least in this experiment, a large response, cold and salt treatment produced similar patterns of transcriptome regulation. Hormone signaling cascades, response to stress markers, lignin biosynthesis, oxidoreductase activity and protein metabolism were induced processes while the whole photosynthetic machinery seems to be halted by these abiotic stress agents.

#### Arabidopsis_IAA treatment dataset

A similar pattern of results was obtained with the larger ATH GeneChip dataset. maSigFun analysis selected relatively few (11) functional categories for which a time and dose effect was significant (Additional file 2). Selected categories had between 3 and 6 annotated genes and an inner correlation value of 0.69 ± 0.15. Regulated functions were *indole-3 acetic acid amido synthetase*, *auxin efflux transporter activity*, *lateral root formation *(Additional file 3). These processes represent the basic response to IAA treatment: the hormone uptake, metabolism and its effects on growth, and showed a maximum induction at 1 hours and 3 hours which was also most pronounced at the higher IAA dose (Additional file 4). Other significant categories were *gibberelin3-beta-dioxygenase activity *and *gibberelin-20 oxidase activity *which were only induced at 1 h, while the *sulfate adenylyltransferase (ATP) activity *and *cyclopropane-fatty-acyl phospholipids synthase activity *showed a down-regulation pattern. The PCA-maSigFun analysis of these data revealed, as expected, the more detailed picture of the functional aspects of auxin treatment. This method selected 92 functional classes (Additional file 2) including GO terms describing the auxin metabolism -*indole-3 acetic acid amido synthetase auxin polar transport*, *response to auxin stimulus*, *auxin:hydrogen symporter activity, auxin mediated signaling pathway*-, the (meristematic) growth -*cell morphogenesis*, *cell-wall modification*, *regulation of meristem size*, *root hair elongation*- and other regulatory and enzymatic activities such as *transcription factor activity*, *ligase activity*, *protein serine/threonine phosphatase activity *(early induction) and *amino acid transporter*, *pectin esterase inhibitor activity*, *proteasome complex*, *oxidorreductase activity *and *beta-fructofuranosidase activity *(late induction). Interestingly PCA-maSigFun shows a regulation of the class *response to water deprivation *which corresponds to repression of plasma membrane aquaporin genes (Additional file 5). Aquaporins mediate hydraulic connectivity across membranes and although water uptake would be concomitant to growth the actual downregulation of aquaporins by auxin treatment has been recently demonstrated in strawberry [42]. Another interesting functional pattern revealed by PCA-maSigFun corresponds to the class *auxin:hydrogen symporter activity*. The function shows a dose-dependent regulation at 1 hour with partial recovery at 3 hours. This regulation is positive for three proteins of the auxin efflux carrier family (PIN3, PIN7 and PIN4) and negative for other class members, At2g17500 and Atg76520 (Additional file 5). The auxin efflux carrier is a membrane system that regulates auxin transport whose polarity responds to the cellular environment [43]. The opposite regulation of members of this complex might reflect this polarity regulation mechanism. Finally, the ASCA-functional methodology applied to the Arabidopsis dataset provided the same basic functional profile. In agreement with the other methodologies, ASCA showed as major pattern of gene expression variation in this dataset the dose-dependent differential regulation at time 1 hours with a slight recovery at time 3 hours which accounted or 77% of the variation associated to the time and treatment factors (Additional file 5). FatiScan analysis on this principal component detected 309 functional classes. Many of the ASCA functional detected classes were semantically related to those obtained by PCA-maSigFun. However, additional GO terms were selected by the ASCA method, mainly corresponding to general functions such as *ribosome*, *thylakoid*, *cytoskeleton*, transferase, isomerase and oxidoreductase activities, possibly revealing the global biological impact of the growth hormone IAA in the plant (Additional file 2).

### Comparison to other methods

Both experimental datasets were additionally analyzed with the STEM software and by the method described by Minguez *et al *[40]. STEM works by assigning gene profiles to predefined clusters and evaluating the significance of the clusters which can then be functionally interrogated by GO enrichment analysis. On the contrary, Minguez *et al. *proposed a methodology whereby pair-wise gene expression differences between time points are computed and used as gene ranking criterion to perform multiple GSAs.

The first problem encountered when using any of the alternative methods was the difficulty in analyzing multiple series data. In the case of STEM this option was simple outside the scope of the methodology while in the case of the pair-wise method considering multiple series would have implied a large number of pair-wise analyses. We therefore defined single series datasets to run comparisons: the high BB dose in case of the toxicogenomics dataset and the salt stressor for the potato study. For the Arabidopsis data two series were defined: one corresponding to the time-effect, by averaging gene expression values for the two IAA doses at each time point (time series), and one for the difference between low and high indole-acetic acid (treatment series).

By running STEM with default parameters on the two one-series datasets a number of significant genes and clusters were found in each case: 253 genes/10 clusters for the high bromobenzene series, 102 genes/3 clusters for the salt treatment, 10078 genes/6 clusters for the time series in the Arabidopsis study and 1971 genes/4 clusters for the treatment series in the ATH data (Table 2 and Additional file 3). However, when performing GO enrichment analysis for the gene sets contained in each significant cluster, no or very few significant functional terms could be obtained in the datasets. Only in the case of the Arabidopsis study a functional result was obtained: the time series indicated 45 GO categories as significant, which mainly consisted of general function such as *chloroplast*, *structural constituent of ribosome *and *membrane*, while the treatment series solely detected the *response to auxin stimulus *as enriched. A closer look to the STEM results revealed that several GO terms did have significant single test *p. values *but not when adjusted by FDR, and that significant clusters had related profiles. This suggests that the limitations of the method to report significant functional classes might be related to the corrections imposed by the multiple testing scenario and/or by a functionally suboptimal data partitioning.

In contrast, the pair-wise method did obtain significant functional results. In total 49 classes were found with the toxicogenomics dataset, 46 were significant for the potato stress study and 172 in the Arabidopsis dataset (Table 2 and Additional file 4). In general, the functional activations portrayed by this method were contained in that learned by the new methodologies. However, some differences were also found. For example, in the case of the potato study, the pair-wise algorithm identified the repression of *glycolsyis *and *gluconeogenesis *at 3 hours post-stress, which was not observed by any of the proposed methodologies. In contrast, our methods revealed numerous enzymatic activities, hormone signaling cascades and tissue developmental processes which were absolutely transparent to Minguez's method. Moreover, the comparison method did not directly indicate the time profile of the identified processes and this information needed to be derived a posteriori from the joint evaluation of the pair-wise results.

## Discussion

The understanding of the cellular and functional implications of global gene-expression changes measured through microarrays is in many cases the ultimate and most important goal of the biological experiments analyzed by this technology. When the experiment includes a time component, the data has a dynamic nature that needs to be incorporated into the functional analysis. The statistical approaches presented and evaluated in this study try to exploit this dynamic property from different perspectives and offer methods that explicitly focus on coordinative behaviors within the cellular functionality along the time span. This is in contrast to more traditional approaches that require a gene selection method and a partitioning algorithm before reaching the stage of functional assessment. maSigFun is, from the three algorithms proposed, the method that more strongly concentrates in co-expression. By fitting one regression model on the expression data gathered by each functional class, it follows that class members need to be highly correlated. Conceptually, maSigFun could be related to the *globaltest *developed by Goelman and co-workers [44] where one statistical model is fitted for a gene set, although the way the two methodologies are carried out is very different. While the *globaltest *treats genes in the set as the dependent variables of the model, maSigFun regresses on experimental factors (time and treatment) and considers individual genes as observations of the values that time and treatment take for the functional class. The simulation studies indicated that only classes with a high proportion of coordinately changing genes (~70%) were readily detected by this method. The experimental datasets confirmed this tendency and also showed a bias in class selection for those with a reduced number of annotated genes and a relatively high (~60%) inner correlation. This is not surprising since large – and frequently more general – functional classes are more likely to include different regulation patterns and to capture more noise. The consequence is that this method is able to reveal specific cellular functionalities which are affected by the experimental conditions but may escape to other interesting phenomena which are not so well defined by a one-block behavior of the functional class. This, which might be sufficient in some cases, may imply a partial result in others where a broader view of the transcriptional changes is sought. In the case of the toxicogenomics dataset maSigFun analysis provided a clearly limited result. Although some detected functions such as *heme oxygenase activity *and *bile acid transporter activity *are key makers of the toxicological response [36], many other important processes such as the *xenobiotic metabolic process, acute-phase response *and *epoxide hydrolase activity *did not show up in this analysis. In the case of the abiotic stress study, however, maSigFun analysis did already provide quite an extensive functional view of the regulated processes, possibly due to the involvement of numerous specific enzymatic activities and cellular locations with a low number of annotated genes, and the more extensive transcriptional profiling (~10 k probes) of the potato dataset. On the contrary, for the ATH – IAA treatment study, this method only selected a few functional classes, although these were highly significant for the biological scenario under study (IAA metabolism, auxin transport and growth). In all three datasets maSigFun selected specific terms, with a reduced number of annotated genes which were highly correlated. These results clearly reveal the detection capacity of the method and also show that this is applicable for datasets of different sizes.

The above-mentioned aspect of the broader evaluation of the transcriptional response from a functional point of view is probably best addressed by the PCA-maSigFun method. In this strategy sub-patterns of time-associated changes within each functional class are identified by PCA analysis followed by regression modeling on the principal components. PCA-maSigFun provided the largest GO term selection in both experimental datasets and the simulated study indicated that the method is able to identify any functional group in which some correlation structure is present. The method should not be considered as an enrichment analysis strategy, but more a methodology to dissect and investigate how genes, functions and co-expression relate. This exercise can be very interesting in some cases, such as in the acute-phase example shown in the toxicogenomics section. Here, PCA-maSigFun clearly showed the correlation and anti-correlation relationships between acute-phase positive and negative genes, which would presumably result in an activation of the process. Another example of this was the class auxin:hydrogen symporter activity in the Arabidopsis data, where also induction and repression of different membrane proteins was observed. Methods that concentrate only in shared profiles would fail to identify these classes in which co-regulation is clearly present. Possibly recently-introduced term relationships in Gene Ontology (*regulates_positively *and *regulates_negatively*) (see ) would help to consider these situations more formally, but to our knowledge there are no functional assessment methods yet that incorporate these relationship descriptors. It is also important to indicate that although PCA-maSigFun is not an enrichment method, it does not return just any functional class. Firstly, PCA assures that selected categories must contain a structure of correlation above the level of noisy variance of each particular dataset and secondly, the maSigPro analysis on the selected components means that these patterns can be fitted to a time-dependent model. In fact, in most of the selected functional terms the significant profile corresponded to the first component of the PCA analysis of the class (data not shown). This implies that the major function-dependent patterns of variation also corresponded to time-related events and consequently are consistent with the biological scenario investigated by the time-course experiment. A possible draw-back of this method is the large size of the resulting selections. This means that browsing the analysis results could be time consuming and that some too general-low informative classes may "artificially" enlarge the output. We partially solved this problem by including only non-annotation redundant GO terms in the analysis (a GO term is considered annotation redundant if it has the same set of annotated genes as any of its child terms). Other options would be to filter results according to the GO structure (by level, by branch, most specific term, etc) or to group significant functional patterns by some clustering method. The last option was implemented in the PCA-maSigFun method and is included in the standard output.

An intermediate result between the restricted view of maSigFun and the profusion of classes given by PCA-maSigFun is obtained by ASCA-functional. In contrast to the two previous methods, this strategy does not imply a transformation from a gene profile to a class profile, but simply ranks genes according to a pattern of variation and assessing a functional enrichment along this rank. This pattern of variation is provided by the ASCA-genes model and, although in this work this is related to time series analysis, the method is generally applicable when more than two conditions are present in the study. In this sense ASCA-functional can be considered as an extension of GSA to multi-class and time series data. Other adaptations of the GSA methodology propose the employment of diverse statistics such as linear modeling and/or posterior probability to measure the association of the gene expression with the phenotype [45], but to our knowledge no statistics have yet been suggested to consider dynamic data. The simulation study indicated that our strategy can identify classes from an inner co-expression level of 50% – 60%, which is indeed in between the other two methodologies presented. ASCA-functional does not provide a detailed analysis of co-expression as in PCA-maSigPro, but it does very naturally show the relationship between functional classes: as the rank provided by the gene loadings in the principal components of the ASCA submodels is a measure of how well each gene follows the pattern identified as major time-dependent expression trends, functional classes overrepresented in the upper part of the rank will follow this pattern while enriched terms at the bottom positions will have the opposite profile. Another particularity of this method is that it only reaches major expression trends, since the PCA models simplify data by their predominant structures. We argue that this, which could be suggested as a limitation for a gene-centric analysis, is of little relevance when considering functional blocks with coordinated behaviors. Recently, [46] proposed a methodology for gene set enrichment analysis based on PCA. However, their approach is very different to ours since the authors use PCA to select gene sets whose one-component projection best associates to the phenotype, rather than to quantify the relationship of individual gene profiles to a defined generic pattern.

## Conclusion

We can conclude that the methodologies presented in this paper are valuable and offer different approaches to study microarray time series data from a functional perspective. The methods should not be considered as competitive but as providing different insights into the molecular and functional events taking place within the biological system under study.

## List of abbreviations used

d.e.g: differentially expressed genes; ANOVA: Analysis of Variance; PCA: Principal Component Analysis; SCA: Singular Component Analysis; GSA: Gene Set Analysis; BB: Bromobenzene; GO: Gene Ontology; BP: Biological Process; MF: Molecular Function; CC: Cellular Component; FDR: False Discovery Rate

## Competing interests

The authors declare that they have no competing interests.

## Authors' contributions

MJN helped to conceive the study, performed simulation studies and helped draft the manuscript, PS performed STEM analysis, ST performed FatiScan analysis, FG-G performed analysis with developed algorithms on experimental datasets, JD contributed to design study and provided research infrastructure, AF supervised statistical developments and helped draft the manuscript, AC conceived and coordinated the study, wrote statistical algorithms and drafted the manuscript.

## Supplementary Material

Additional file 1 Results simulation studies for the three proposed methods. Click here for file

Additional file 2 Gene Ontology term selection by the three proposed methods on three experimental datasets. Click here for file

Additional file 3 Clustering results of STEM analysis on three experimental datasets. Click here for file

Additional file 4 Gene Ontology term selection by the pair-wise methods on three experimental datasets. Click here for file

Additional file 5 Selected gene expression patterns for the functional analysis of the Arabidopsis IAA treatment study. Click here for file
