# Supplementary material for: Functional assessment of time course microarray data
Source: BMC Bioinformatics. 2009 Jun 16;10(Suppl 6):S9. doi: 10.1186/1471-2105-10-S6-S9 (PMC2697656; doi:10.1186/1471-2105-10-S6-S9)
Supplement: Additional file 5 — Selected gene expression patterns for the functional analysis of the Arabidopsis IAA treatment study. [file 1471-2105-10-S6-S9-S5.pdf]

maSigFun analysis of Arabidopsis indole-acetic acid dataset.
Indole-3-acetic acid amido synthetase class

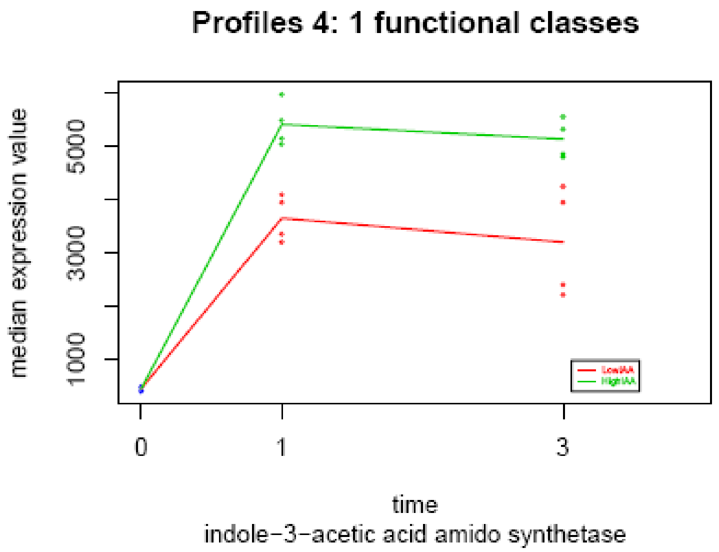

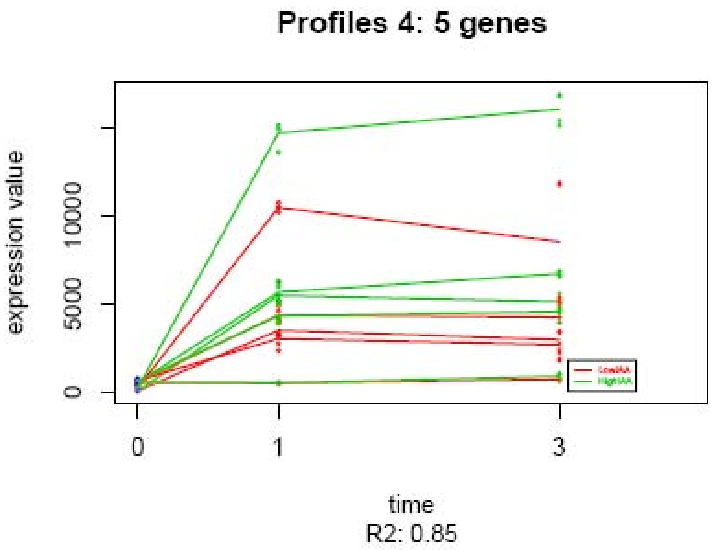

Left panel panel shows the median profile of the class, correspondig to the upregulation of gene expression at time points 1 hour and 3 hours, which is more pronounced at the high IAA dose. The right panel shows the indivdual expression profiles of the 5 genes annotated to this class. The mean pair-wise correlation of these 5 genes is 0.85.

PCA-maSigFun analysis of Arabidopsis indole-acetic acid dataset. *auxin:hydrogen symporter activity* class

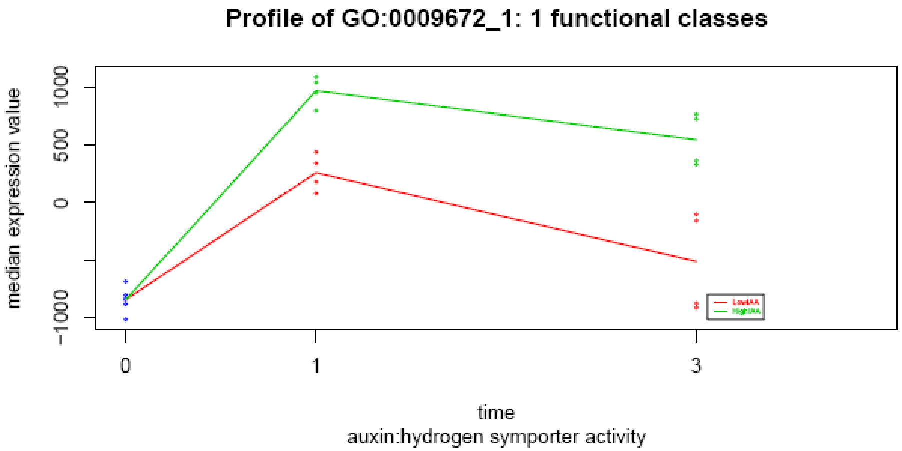

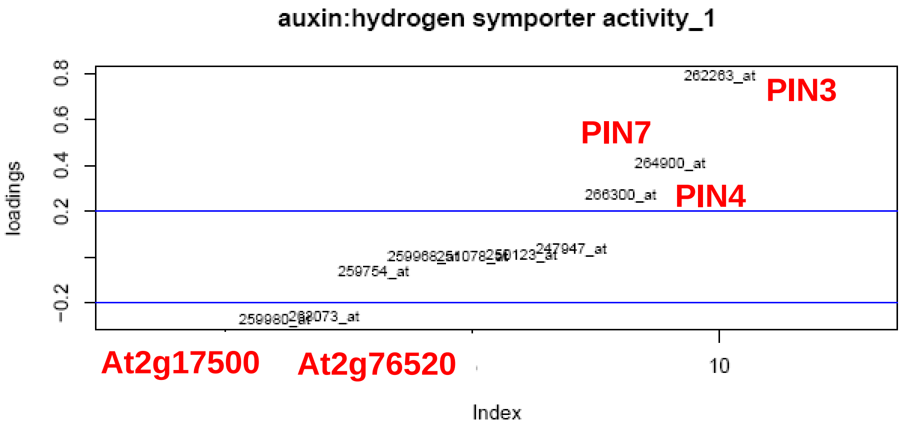

The auxin:hydrogen symporter activity class is bi-modal. Genes of the PIN class (PIN3, PIN4 and PIN7) are positively correlated (positive loadings, right panel) with the extracted class pattern, i.e. dose-dependent induction at time point 1hours (left panel). Two other genes (the auixn effux carrier proteins At2g17500 and At2g76520) show negative correlation (negative loadings) with the class pattern, i.e., are repressed at time point 1 hour.

ASCA functional analysis of Arabidopsis indole-acetic acid dataset. Score plot of Principal Component 1 of time x treatment submodel

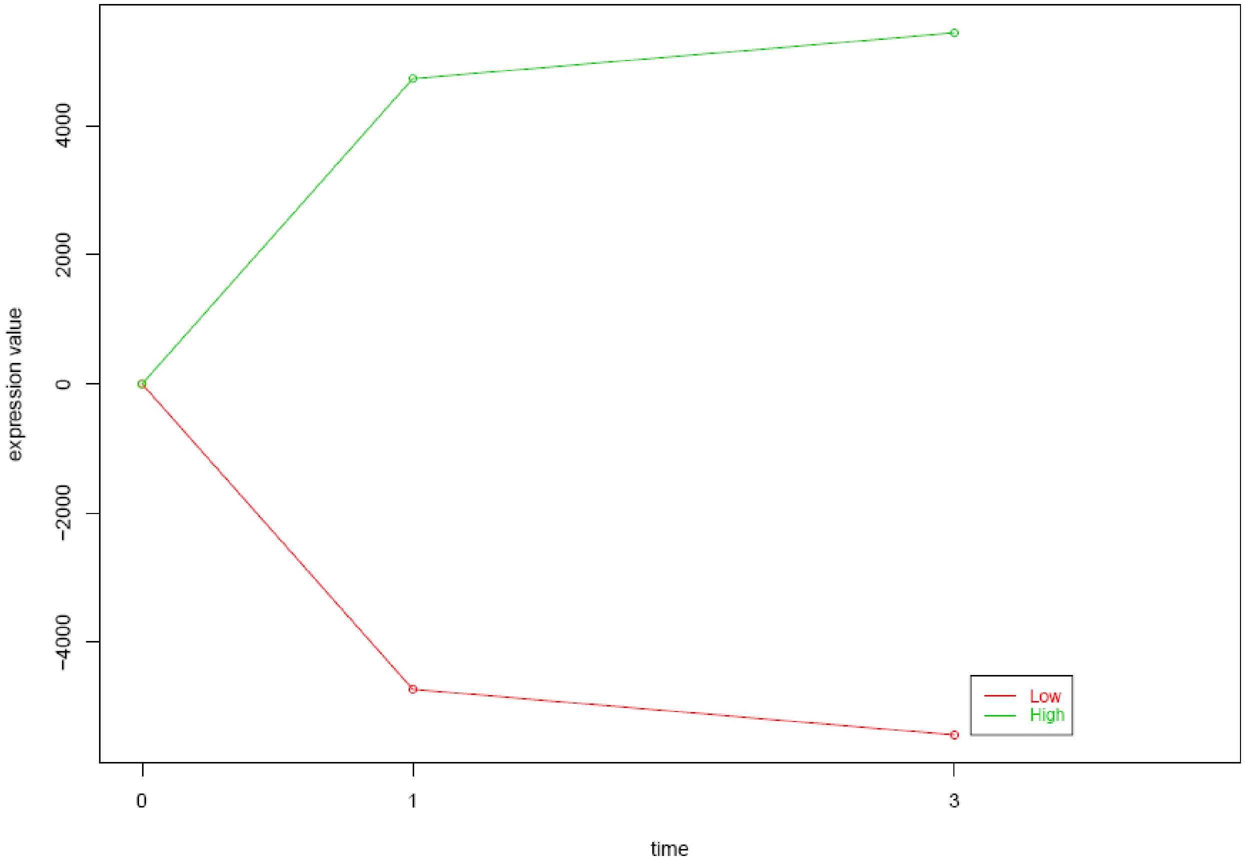

The first principal component (77% explained variability of the time x treatment interaction) describes the different expression level at time point 1hours and 3 hours between low and high IAA doses
